# DNA Methylation Regulates CDK5R1 and NRBP1 to Exert Effects on Alcohol Dependence: Insights From Mendelian Randomization

**DOI:** 10.1111/adb.70162

**Published:** 2026-04-23

**Authors:** Fuyuan Deng, Junsheng Peng, Siran Lai, Guangpeng Zhang, Miaomiao Li, Yuxin Huang, Mi Yuan, Gaolei Yao, Peiming Zhang, Liming Lu

**Affiliations:** ^1^ Clinical Research and Big Data Laboratory, South China Research Center for Acupuncture and Moxibustion, Medical College of Acu‐Moxi and Rehabilitation Guangzhou University of Chinese Medicine Guangzhou China; ^2^ The Second Clinical Medical College Guangzhou University of Chinese Medicine Guangzhou China; ^3^ The First Clinical Medical College of Guangzhou University of Chinese Medicine Guangzhou China; ^4^ Department of Radiology The First Affiliated Hospital, Sun Yat‐Sen University Guangzhou China; ^5^ School of Medical Imaging Guizhou Medical University Guiyang China; ^6^ Foshan Hospital of Traditional Chinese Medicine Affiliated to Guangzhou University of Chinese Medicine Foshan Guangdong China

**Keywords:** alcohol dependence, DNA methylation, druggable genes, mendelian randomization

## Abstract

Alcohol dependence currently lacks targeted pharmacotherapies, underscoring the urgent need for novel therapeutic targets. Existing research on disease‐associated DNA methylation changes and their gene regulatory effects remains inconsistent. To resolve this uncertainty, we applied the Mendelian randomization to elucidate causal mechanisms connecting druggable genes, epigenetic regulation and alcohol dependence development. Integrating MR, colocalization and mediation analyses, we leveraged genome‐wide association study (GWAS) (FinnGen), eQTL (eQTLGen) and methylation (GoDMC) data. We assessed causal gene‐alcohol dependence relationships, shared causal variants via colocalization and methylation‐mediated regulatory mechanisms. Our integrative analysis identified 10 drug‐targetable genes showing significant expression alterations in alcohol dependence (FDR < 0.05), with three genes (CDK5R1, CAMKK2 and NRBP1) demonstrating evidence of shared causal variants through colocalization. Epigenetic regulation was particularly evident at two methylation sites (cg07437263 and cg05102552) that indirectly influenced alcohol dependence risk by modulating CDK5R1 (63.92% mediation) and NRBP1 (95.12% mediation) expression. These findings reveal DNA methylation as a critical regulatory mechanism governing neuronal gene expression patterns in alcohol dependence pathogenesis. The strong mediation effects observed for CDK5R1 and NRBP1, coupled with their colocalization evidence, position these genes as promising candidates for both biomarker development and targeted therapeutic interventions in alcohol dependence. This investigation spotlights the regulatory function of DNA methylation on CDK5R1 and NRBP1 in alcohol dependence. It implies that CDK5R1 and NRBP1 could serve as potential clinical biomarkers or therapeutic targets for the early management of alcohol dependence.

## Graphical abstract

This study employed integrative Mendelian Randomization analysis to identify 10 drug‐targetable genes with altered expression in alcohol dependence. Colocalization evidence suggests shared causal variants. Crucially, DNA methylation at specific sites (cg07437263, cg05102552) mediates risk by upregulating the protective gene CDK5R1 (63.92%) and dysregulating the risk gene NRBP1 (95.12%).

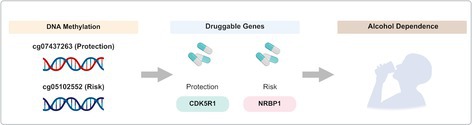

**Abbreviations**
ALS: amyotrophic lateral sclerosis
AUD: alcohol use disorder
CDK5: the cyclin‐dependent serine/threonine kinase 5
cis‐eQTLs: cis‐expression quantitative trait loci
GWAS: genome‐wide association study
IVs: instrumental variables
IVW: inverse‐variance weighted
MAOA: monoamine oxidase A
MR: Mendelian randomization
NAc: nucleus accumbens
OR: odds ratio
VTA: ventral tegmental area

## 1 Introduction

Alcohol dependence is a chronic and frequently relapsing disorder characterized by persistent alcohol use despite negative consequences. In 2016, approximately 104 million cases were reported globally. Furthermore, alcohol dependence accounted for a total of 99.2 million disability‐adjusted life years (DALYs) worldwide, resulting in substantial socio‐economic losses [1, 2]. The clinical management of alcohol dependence currently faces the dual challenges of limited treatment options [3] and high relapse rates [4, 5]. Traditional drug discovery paradigms are often protracted, costly and exhibit low success rates. In this context, drug repurposing has gained prominence as a strategy capable of significantly shortening development timelines and reducing costs [6].

The rapid advancement of genomic research has yielded extensive genome‐wide association study (GWAS) catalogues, which provide valuable resources on potentially druggable genetic targets associated with alcohol dependence [7]. Concurrently, epigenetics research, particularly on DNA methylation—a key regulator of gene expression—has revealed strong links with alcohol dependence [8]. This confluence offers an opportunity to integrate multi‐omics approaches, combining genetics and epigenetics, to advance precision medicine for alcohol dependence [9].

This study proposes a systematic research strategy: First, utilize GWAS data to identify druggable genes significantly associated with alcohol dependence. Second, apply colocalization analysis to identify shared genetic loci between these druggable genes and alcohol dependence, ensuring robust biological relevance. Subsequently, identify upstream DNA methylation sites that have a causal relationship with both the druggable genes and alcohol dependence. Finally, employ the Mendelian randomization (MR) methods to establish the causal direction from DNA methylation to druggable gene expression to alcohol dependence, thereby constructing a comprehensive pathogenic pathway.

## 2 Methods

### 2.1 Study Design

**FIGURE 1:**
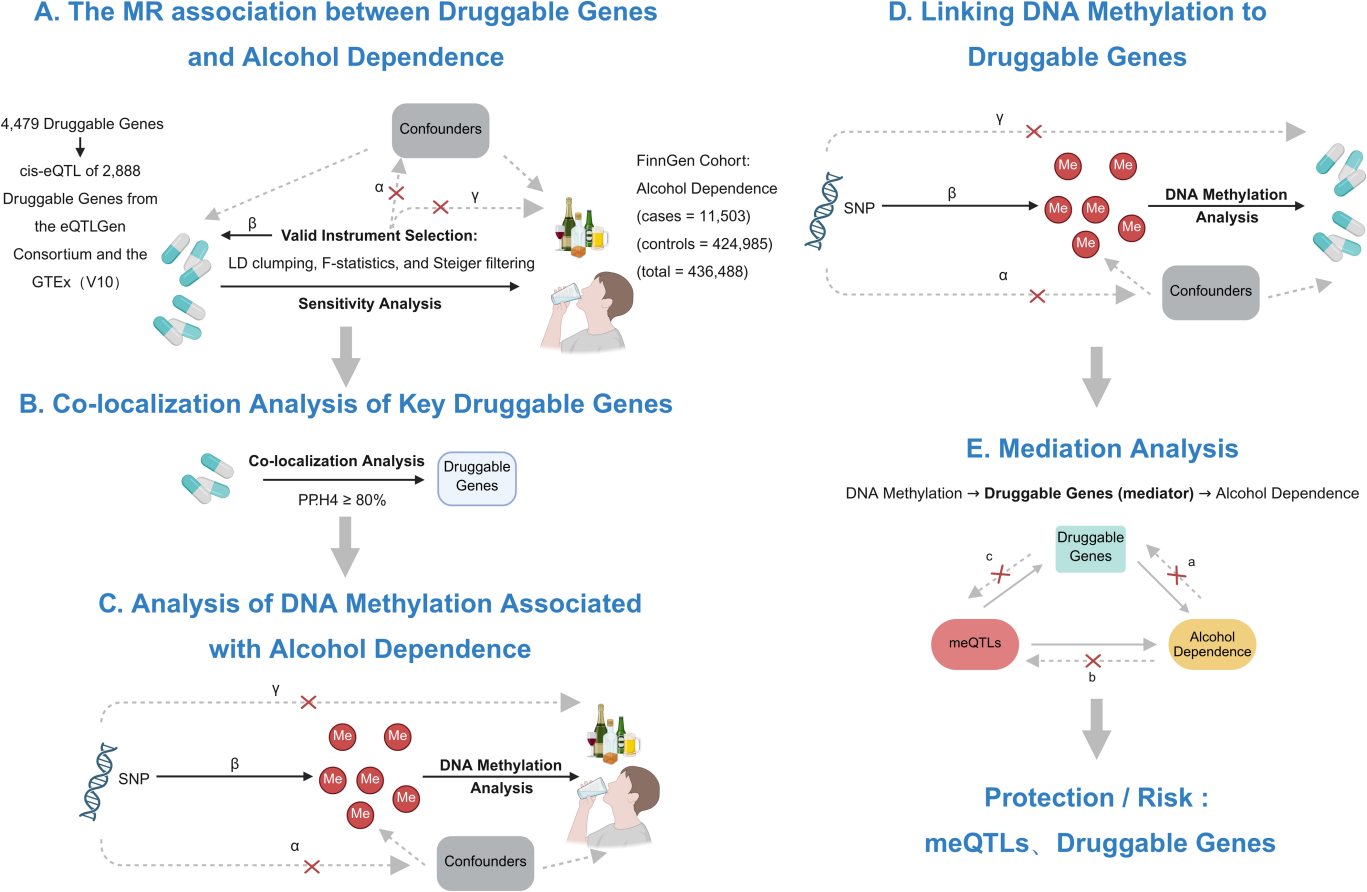
The flowchart of our study design. Legend: (A) MR analysis of the association between druggable genes and alcohol dependence. Here, *α* represents the independence assumption, *β* denotes the association assumption and *γ* indicates the exclusion assumption. The selection of instrumental variables was based on the criteria: *p*‐value < 5 × 10^−8^, *r* ^2^ < 0.1 and *kb* = 10 000. (B) Co‐localization analysis of key druggable genes. (C) Analysis of DNA methylation associated with alcohol dependence, where *α*, *β*, and *γ* are defined as above. (D) Linking DNA methylation to druggable genes. (E) Mediation analysis, where *a*,*b* and *c* indicates the absence of reverse MR.

Figure 1 outlines the analytical workflow. First, blood‐derived cis‐expression quantitative trait loci (cis‐eQTLs) of druggable genes are used as the exposure, and GWAS data for alcohol dependence as the outcome, to identify druggable genes significantly associated with alcohol dependence. Second, colocalization analysis is applied to identify druggable genes for which the cis‐eQTLs and alcohol dependence GWAS share a common causal variant, ensuring the reliability of the genetic association. Subsequently, using methylation quantitative trait loci (metaQTLs) of the identified druggable genes (those significantly associated with alcohol dependence and sharing a common causal variant) as the exposure and the blood‐derived cis‐eQTL data of these druggable genes as the outcome, upstream DNA methylation sites for these genes are identified. Finally, a MR analysis is conducted using these upstream methylation sites (associated with the significant, colocalized druggable genes) as the exposure and the alcohol dependence GWAS data as the outcome, to confirm a significant causal relationship between the methylation sites and alcohol dependence. The entire protocol employs a mediation MR approach to clarify the causal direction from DNA methylation to the druggable gene and subsequently to alcohol dependence, thereby constructing a complete pathogenic pathway.

### 2.2 Data Source

From Finan et al.'s compendium of 4479 druggable genes [7], we extracted blood‐derived cis‐eQTLs for druggable genes from the eQTLGen Consortium [10](https://eqtlgen.org/) and the GTEx (V10) (https://gtexportal.org/). The dataset used for alcohol dependence was sourced from FinnGen Release 11, which became publicly available in June 2024 and can be found online (https://www.finngen.fi/en). The dataset, designated as finngen_R11_F5_ALCOHOL_DEPENDENCE.gz, encompasses 11 503 individuals with alcohol dependence and 424 985 control samples. DNA methylation sites data were obtained from the GoDMC database, comprising genome‐wide meta‐analyses of 420 509 CpG sites. The complete dataset is publicly available through the GoDMC repository (http://mqtldb.godmc.org.uk/downloads). DNA methylation sites for druggable genes were retrieved from NGDC (https://ngdc.cncb.ac.cn/ewas/datahub/exploration). All data were acquired in accordance with original study protocols, including participant consent and ethical approvals. Detailed information of each database can be found in Table 1. Currently, no studies have found any sample overlap between the GoDMC database and the eQTLGen consortium, GTEx (V10) or the Finnish database.

**TABLE 1:** Sample size, racial ancestry samples of the four database [10, 11, 12, 13].

Database	Sample size	Racial ancestral background
GTEx v10	838 cases	American ancestry
eQTLGen Consortium	31 684 cases	European ancestry
FinnGen 11	436488cases	Finnish ancestry
GoDMC	27 750 cases	European ancestry

### 2.3 MR Analysis of the Relationship Between Druggable Genes in the eQTLGen

#### 2.3.1 MR Analysis of the Relationship Between Druggable Genes in the eQTLGen Consortium and Alcohol Dependence

Causal relationships between genetically proxied druggable gene expression and alcohol dependence were evaluated through two‐sample MR analyses implemented in the ‘TwoSampleMR’ R package (version 0.5.7). Expression‐associated single nucleotide polymorphisms meeting genome‐wide significance thresholds of *p*‐value less than 5 × 10^−8^ and demonstrating strong instrument strength with *F*‐statistics exceeding 20 were selected as instrumental variables [14] after pruning for linkage disequilibrium (LD; *r* ^2^ < 0.1 within 10 000 kb) [15]. For single‐SNP genes, we applied the Wald ratio method; multi‐SNP genes were analysed using inverse‐variance weighted (IVW) regression as primary analysis, supplemented by MR‐Egger, weighted median and mode‐based estimators [16]. Effect sizes represent the odds ratio (OR) for alcohol dependence per 1‐SD increase in predicted gene expression. We implemented Steiger filtering to confirm correct causal direction (exposure variance > outcome variance) and repeated primary analyses after excluding variants failing this assumption (Tables S2 and S3).

#### 2.3.2 MR Analysis of the Relationship Between Druggable Genes in the GTEx(V10) and Alcohol Dependence

Due to the relatively small number of SNPs included in the GTEx (V10) database, we separately selected druggable genes with *p* < 5 × 10^−5^, LD: *r* ^2^ < 0.1 within 10 000 kb and LD: *r* ^2^ < 0.3 within 10 000 kb for the MR analysis with alcohol dependence. The specific operation is the same as that of the MR analysis of druggable genes and alcohol dependence in the eQTLGen Consortium.

### 2.4 Sensitivity Analysis

We performed multiple sensitivity analyses to ensure result reliability. First, Cochran's *Q* test evaluated instrumental variable heterogeneity, with nonsignificant results (*p* > 0.05) indicating acceptable homogeneity. Second, we assessed potential pleiotropic effects through MR‐Egger regression, where an intercept *p*‐value exceeding 0.05 suggested minimal directional pleiotropy [17]. Third, the MR‐PRESSO approach identified and corrected for outlier variants potentially affected by pleiotropy, with the global test *p* > 0.05 supporting model validity. Finally, leave‐one‐out analysis systematically examined each variant's influence by iteratively excluding individual instruments and re‐estimating effects.

### 2.5 Colocalization Analysis

We conducted Bayesian colocalization analysis for drug‐targetable genes showing significant MR associations using the R package ‘coloc’ (v5.2.1) [18] with default priors (P1 = P2 = 1 × 10^−4^, P12 = 1 × 10^−5^). This evaluated five competing hypotheses regarding shared genetic architecture between gene expression and alcohol dependence: PPH0 (no association with either trait), PPH1 (expression‐associated only), PPH2 (outcome‐associated only), PPH3 (separate causal variants for both traits) and PPH4 (shared causal variant; PPH4 > 0.80 considered significant evidence [19]). The analysis focused on the lead exposure‐associated variant (most significant SNP) and neighbouring variants within ± 500 kb. Genes meeting the PPH4 threshold were identified as high‐confidence candidates for alcohol dependence, suggesting potential therapeutic targets through shared causal mechanisms.

### 2.6 Mediation Analysis of DNA Methylation‐Druggable Genes‐Alcohol Dependence Relationship

We performed integrated two‐sample and mediation MR analyses [20] to examine (1) the causal effect of DNA methylation on alcohol dependence, (2) the causal effect of DNA methylation on druggable genes and (3) the causal effect of druggable genes on alcohol dependence, thereby elucidating the pathogenic pathway through which DNA methylation influences alcohol dependence via the regulation of druggable genes. The total effect was decomposed into path c (βc, representing the total effect of DNA methylation on alcohol dependence) and the indirect effect (βa × βb, mediated by the pathway DNA methylation → druggable gene → alcohol dependence). The direct effect was calculated as βc—βa × βb, and the mediation proportion was quantified as (βa × βb) /βc [20]. Through this mediation MR analysis, we quantified the contribution of druggable genes to the association between DNA methylation and alcohol dependence. Additionally, we conducted sensitivity analyses to verify the robustness of the findings.

### 2.7 Statistics

All analyses were performed in R (version 4.3.2) with the TwoSampleMR package (version 0.5.7) and the MR‐PRESSO package (version 1.0). We performed FDR correction on the results. The adjusted *p*‐values less than 0.05 were considered statistically significant. Meanwhile, *p* < 0.05 was regarded as nominally significant.

## 3 Results

### 3.1 MR Analysis of the Relationship Between Druggable Genes in the eQTLGen Consortium and Alcohol Dependence

Based on predefined criteria, genome‐wide analysis yielded 40 356 SNPs meeting criteria as valid instrumental variables for 2888 druggable genes. All chosen IVs demonstrated an *F*‐statistic greater than 20, suggesting the absence of any weak instrument bias. Comprehensive details regarding these instrumental variables can be located in the Supporting Information: Table S3. Importantly, consistency was observed in the directional effects of all 20 genes across the three methodologies applied (refer to Tables S4.1–S4.2). In addition, the application of Steiger filtering validated the relationship between druggable gene expression and the status of alcohol dependence (Tables S7–S9).

**FIGURE 2:**
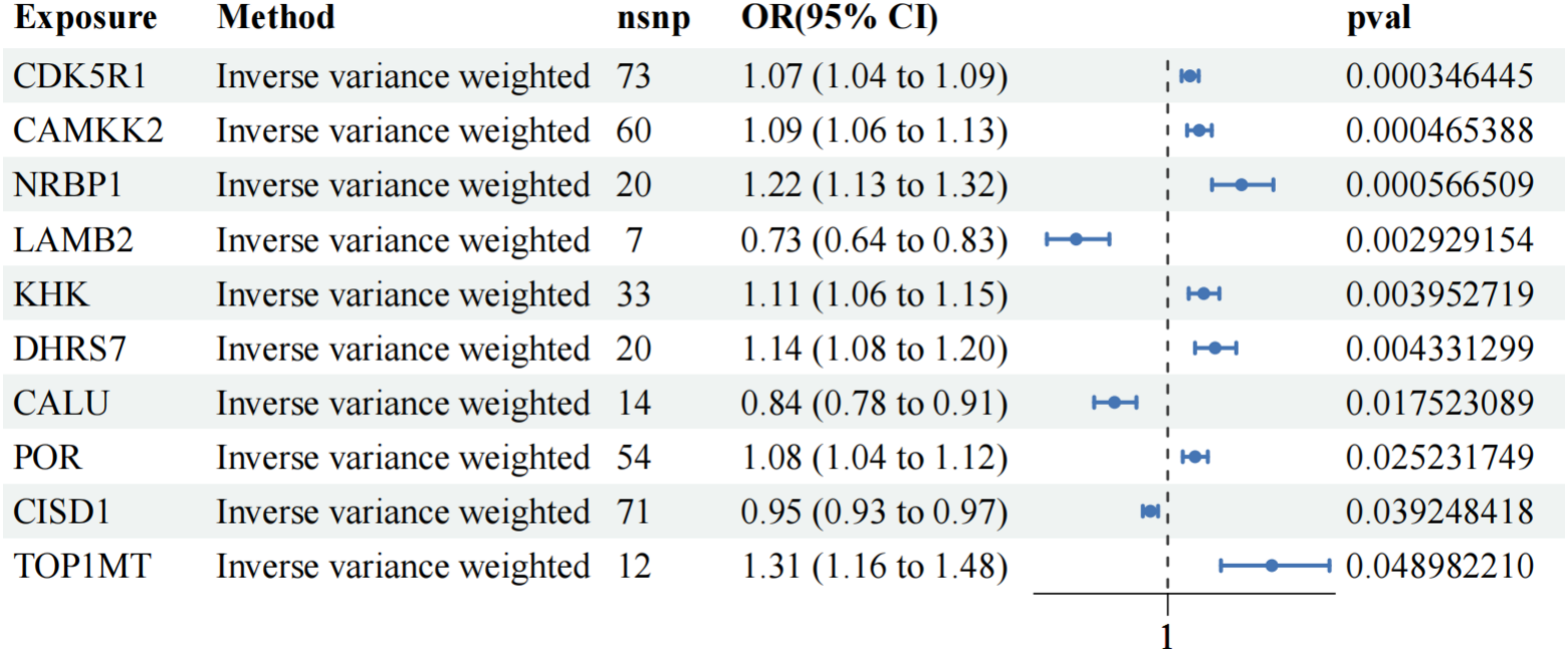
The FDR‐corrected results of the MR between the eQTL of druggable genes and alcohol dependence.

Using IVW regression, we identified significant associations between three druggable genes' expression and alcohol dependence (FDR < 0.05; Figure 2). The causal estimates demonstrated that elevated expression of CDK5R1, CAMKK2 and NRBP1 was positively associated with increased alcohol dependence risk, suggesting potential pathogenic roles. Figure 2 visually summarizes these IVW‐derived causal relationships.

### 3.2 MR Analysis of the Relationship Between Druggable Genes in the GTEx(V10) and Alcohol Dependence

Replication analysis in the GTEx (V10) database confirmed the significant associations of CDK5R1, CAMKK2 and NRBP1 with alcohol dependence previously identified in the eQTLGen Consortium. Applying stringent thresholds (*p* < 5 × 10^−5^, kb < 10 000), IVW analysis revealed significant associations for NRBP1 (*r* ^2^ < 0.3; OR = 1.242, 95% CI: 1.074–1.436, *p* = 3.376 × 10^−3^) and CDK5R1 (*r* ^2^ < 0.1; OR = 1.215, 95% CI: 1.101–1.341, *p* = 1.011 × 10^−4^). For CAMKK2, which contained only one eligible SNP, Wald ratio analysis demonstrated a particularly strong association (OR = 1.952, 95% CI: 1.300–2.930, *p* = 1.239 × 10^−4^) (Tables S10–S12).

### 3.3 Colocalization Analysis

Colocalization analysis of significant MR‐associated druggable genes revealed strong evidence (PP.H4 > 80%) that alcohol dependence shares causal variants with three genes: CDK5R1 (82%), CAMKK2 (84%) and NRBP1 (97%). These findings, supported by both MR and colocalization evidence (Table S5), suggest that CDK5R1, CAMKK2 and NRBP1 may promote alcohol dependence risk and represent promising therapeutic targets.

### 3.4 Mediation Analysis of DNA Methylation‐Druggable Genes‐Alcohol Dependence Relationship

**FIGURE 3:**
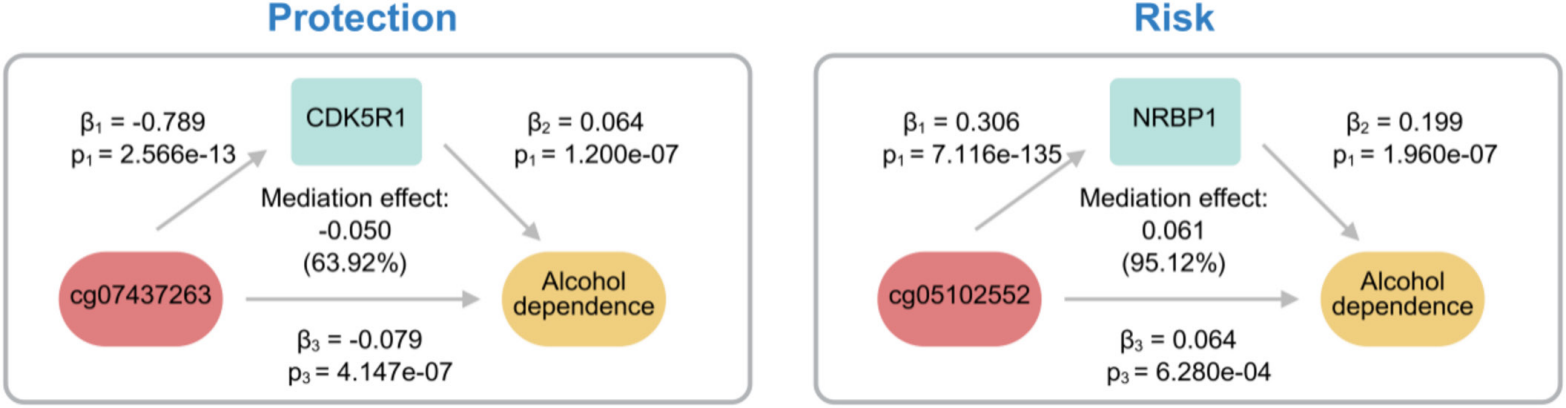
Mediation model of DNA methylation effects on alcohol dependence via CDK5R1 and NRBP1 expression.

We obtained methylation site data for CDK5R1 and NRBP1 from NGDC and GoDMC databases. Initial analysis evaluated DNA methylation effects on gene expression (pathway a, βa) (Figure 3). To ensure robustness, we excluded methylation sites with single SNPs (CDK5R1: cg21855910, cg19634527, cg06366062, cg03898962; NRBP1: cg15478930), retaining six SNPs as IVs (Tables S13 and S16). IVW analysis revealed significant associations: cg07437263 negatively regulated CDK5R1 expression (OR = 0.400, 95% CI: 0.299–0.534, *p* = 5.787e‐10), while cg05102552 positively influenced NRBP1 expression (OR = 1.358, 95% CI: 1.325–1.391, *p* = 7.116e‐135). These methylation sites also showed significant MR effects on alcohol dependence: cg07437263 (OR = 0.923, 95% CI: 0.895–0.953, *p* = 4.147e‐07) and cg05102552 (OR = 1.066, 95% CI: 1.027–1.106, *p* = 6.286e‐04). Two methylation sites (cg07437263 and cg05102552) promoted alcohol dependence progression predominantly via CDK5R1 (63.92%) and NRBP1 (95.12%) expression modulation, as quantified through mediation analysis. Complete mediation results are shown in Figure 3 (detailed data in Tables S13–18).

### 3.5 Sensitivity Analysis

Sensitivity analyses evaluating instrument bias revealed distinct patterns across genes. For CDK5R1 and NRBP1 in the eQTLGen Consortium, we observed no significant heterogeneity or pleiotropy, with consistent effect directions across analytical methods. In contrast, CAMKK2 analysis showed significant heterogeneity, while no pleiotropy was detected (Table S6).

In the GTEx database, we found that the results of NRBP1 showed heterogeneity, but there was no pleiotropy. The results of CDK5R1 and CAMKK2 indicated that there was neither heterogeneity nor pleiotropy.

In the analysis of the relationship between DNA methylation site cg07437263 and druggable gene CDK5R1, heterogeneity was detected, and the specific reasons for the heterogeneity may require further investigation. Since no detection was made in the horizontal pleiotropy test of the DNA methylation site cg07437263 and the drug‐responsive gene CDK5R1, the strong correlation between the DNA methylation site cg07437263 and the gene CDK5R1 can be guaranteed. During the analysis of the DNA methylation site cg05102552, no heterogeneity or pleiotropic effects were found.

## 4 Discussion

We implemented a two‐sample MR study design, colocalization analysis and mediation analyses to evaluate causal connections between druggable genes and alcohol dependence, identifying a significant association between DNA methylation modifications and alcohol dependence risk, mediated by CDK5R1, NRBP1 expression. This study elucidates novel pathogenic mechanisms in alcohol dependence, demonstrating the critical involvement of epigenetic dysregulation in neurodegenerative processes. Our integrated MR and colocalization analyses identified CDK5R1, CAMKK2 and NRBP1 as clinically promising targets, with elevated expression levels showing significant positive associations with disease risk. These molecular markers offer potential for both diagnostic development and therapeutic intervention in early‐stage alcohol dependence.

As a member of the calcium/calmodulin‐dependent kinase family, CaMKK2 functions as a serine/threonine‐specific protein kinase and a critical metabolic regulator through its activation of downstream effector [21]. This kinase demonstrates particularly high expression in neuronal populations, where it modulates both cerebral energy metabolism and higher order cognitive processes such as long‐term memory formation and affective regulation [22]. Emerging evidence implicates CaMKK2 in alcohol‐related neuroadaptations, particularly through its interaction with small‐conductance SK channels (calcium‐activated potassium channels) in key reward circuitry regions including the hippocampus, ventral tegmental area (VTA) and nucleus accumbens (NAc). These K (Ca)2 channels exhibit remarkable sensitivity to both immediate and long‐term alcohol administration, modulating dopaminergic neuron excitability and contributing to withdrawal‐related hyperexcitability and escalated alcohol consumption [23]. Importantly, drug‐induced modulation of SK channel activity has been shown to bidirectionally regulate voluntary alcohol intake in rodent models while ameliorating withdrawal symptoms and associated neurotoxicity [23]. Based on these mechanistic insights, we propose that CaMKK2 overexpression may drive maladaptive changes in K (Ca)2 channel function, thereby promoting increased alcohol self‐administration behaviors through dysregulation of reward circuit activity.

The CDK5R1 gene product, p35, serves as the principal regulatory subunit and specific activator of CDK5 (the cyclin‐dependent serine/threonine kinase 5) [24] and governs synaptic plasticity, dopaminergic signalling and dendritic spine remodelling—processes central to reward learning and drug‐seeking behaviors [25, 26]. Previous studies reveal that CDK5 inhibition attenuates cocaine‐induced behavioural sensitization [27], while its overexpression exacerbates ethanol‐induced neurodegeneration [28]. This is in line with our research findings. This could also be the reason why overexpression of CDK5R1 aggravates alcohol dependence. The latest research has found that the excessive activation of Cdk5 affects Parkinson's disease and Parkinson's dysfunction through oxidative stress, mitochondrial defects and dopaminergic dysfunction [29]. In the neurodegenerative disorder amyotrophic lateral sclerosis (ALS), we have also observed that hyperactivity of Cdk5 has been demonstrated in the spinal cord of the SOD1G93A mouse model of ALS [30]. Observations have shown that excessive activation of Cdk5 may lead to a decline in cognitive function in patients with HIV [31, 32, 33, 34]. These studies indicate that although the disease targets are different, CDK5R1 may share a common mechanism in the functional impairment and neurodegeneration.

Existing studies have shown that overexpression or inhibition of NRBP1 can have an effect on the occurrence of various cancers [35, 36], but there are relatively few studies on the correlation between NRBP1 and alcohol dependence. With the development of GWAS, NRBP1 shows promise as a pharmacological target for diabetes [37], gout [38] and Parkinson's disease [39]. Meanwhile, a multi‐omics MR study [40] on alcohol use disorders and problem drinking conducted MR analysis on eight classic brain cell types and problematic alcohol use. The study found that the overexpression of NRBP1 in inhibitory neurons in the brain could promote the occurrence of problematic alcohol use [40]. However, in the replication cohort study involving the Finnish population, the study did not obtain the result that NRBP1 could promote problematic alcohol use. Instead, it replicated the finding that the overexpression of NRBP1 could reduce alcohol‐related diseases/deaths. For this purpose, we reviewed the ninth round of GWAS data of the Finnish population. The data included GWAS data for the outcome ‘alcohol use disorder’. Therefore, we hypothesized that the overexpression of NRBP1 in inhibitory neurons in the brain did not lead to an increased risk of alcohol dependence in the Finnish cohort. In contrast, the eQTL data of whole blood used in our study were obtained from the eQTLGen Consortium and the GTEx. At the same time, the result showed that the overexpression of NRBP1 did not lead to an increase in the risk of alcohol dependence. The co‐localization analysis indicated that there was a high possibility that they shared common pathogenic variations. Given that the exposures and outcomes used in this study are different from those in the previous MR analysis, our conclusions do not conflict with the above ones. Nuclear receptor binding protein 1 is a widely expressed and highly conserved pseudokinase [29] and a ubiquitin‐related protein that regulates Wnt/β‐catenin signaling [41]. It affects neuronal survival and glial cell activation [35, 42]. Meanwhile, NRBP1 is associated with the process of neural development and synaptic plasticity [43], which is a key pathway in addiction pathology. Specifically, the mechanism by which NRBP1 promotes alcohol dependence still requires further experimental verification.

Through causal mediation analysis, we established that the cg05102552 methylation site exerts indirect effects on alcohol dependence progression via NRBP1 transcriptional regulation, with NRBP1 expression mediating 95.12% of the observed phenotypic variance. In addition, we found that the methylation site cg07437263 of CDK5R1 can reduce the expression of CDK5R1, thereby reducing the risk of alcohol dependence, with 63.92% of the total effect operating through CDK5R1 transcriptional regulation. This also provides a reference for the treatment of alcohol dependence. An investigation analysed peripheral blood DNA methylation patterns in 135 males with alcohol use disorder (AUD) compared to 150 healthy controls [44]. The analysis revealed significantly elevated methylation levels in AUD patients compared to controls, indicating that peripheral blood DNA methylation has significant clinical value for AUD. Existing literature further establishes that alcohol consumption patterns and associated DNA methylation changes exert causal influences on various pathological conditions including psychiatric disorders [45], colorectal cancer [46] and breast cancer [47]. Importantly, cg07437263 and cg05102552 represent previously unreported methylation sites in alcohol dependence research. The epigenetic modifications primarily occur at CpG‐rich promoter regions and have been demonstrated to significantly impact nervous system functionality and environmental response mechanisms [48]. Aberrant methylation patterns in brain reward circuits, such as the prefrontal cortex [49, 50] and striatum [51], have been linked to addictive behaviours by altering synaptic plasticity, neurotransmitter signalling and stress responses. For instance, hypermethylation of MAOA (monoamine oxidase A) in heavy drinkers correlates with reduced enzymatic activity and heightened impulsivity13. These findings position DNA methylation as a critical mediator of alcohol‐induced neuroadaptations, yet causal relationships remain poorly defined.

Our findings suggest that promoter methylation of CDK5R1 and NRBP1 may critically regulate their transcriptional activity and neuronal expression patterns, potentially mediating their functional roles in alcohol dependence. This epigenetic mechanism could represent a novel therapeutic target for intervention. Additionally, the methylation profiles of these genes show promise as potential diagnostic biomarkers for alcohol dependence. Future research should investigate the feasibility of detecting these methylation signatures in peripheral biofluids (e.g., blood or cerebrospinal fluid), which could enable noninvasive screening approaches and facilitate personalized treatment strategies for alcohol dependence.

This investigation offers several notable strengths. First, our application of MR methodology provides robust causal inference while minimizing confounding biases and reverse causality inherent in cohort or case–control analyses. Second, the integration of MR with colocalization analyses and mediation analyses not only confirmed the involvement of CDK5R1, CAMKK2 and NRBP1 in alcohol dependence but also elucidated the regulatory role of DNA methylation modifications. Our multi‐analytical approach significantly enhances the validity and mechanistic insights of our findings. Furthermore, the utilization of large‐scale datasets comprising 11 503 alcohol dependence cases and 424 985 controls from the FinnGen consortium ensures substantial statistical power and generalizability. The incorporation of druggable gene data from multiple independent sources further reinforces the reliability of our conclusions.

Several limitations warrant consideration. Firstly, as for alcohol dependence as the outcome, the use of Finnish data may limit the generalizability of our research results to other racial groups, and it needs to be verified in more diverse cohorts. Therefore, we calculated the Finnish enrichment index (FEI) for each IV of the drugable genes and methylation sites: FEI = allele frequency (Finnish population)/allele frequency (non‐Finnish European population). We conducted SNP enrichment analysis, based on the definition of the FinnGen flagship study [52], and defined variations with FEI > 2.0 as ‘strongly Finnish‐enriched variants’. We found that the effects of SNPs with high Finnish enrichment and those with low Finnish enrichment were the same on the leave‐one‐out plot (Tables S19–22). This has certain significance for the generalization of the conclusion. Second, although our computational analyses yield robust evidence, the lack of experimental validation through in vitro cellular models or animal studies for the identified genes and methylation sites remains a critical limitation. Third, potential environmental factors such as toxin exposure and their interactions with epigenetic mechanisms were not addressed in our study, despite their likely contribution to alcohol dependence pathogenesis, highlighting an important avenue for future research. Finally, the clinical utility of CDK5R1 and NRBP1 methylation patterns as diagnostic biomarkers or therapeutic targets requires verification through large‐scale prospective studies.

## 5 Conclusions

This investigation systematically elucidates the mechanistic role of DNA methylation in modulating CDK5R1 and NRBP1 expression during alcohol dependence progression, leveraging an integrative analytical framework combining MR, colocalization and mediation approaches. The results advance our understanding of alcohol dependence pathogenesis by identifying novel epigenetic regulatory pathways, while also underscoring the broader implications of methylation‐mediated gene regulation in neurodegenerative processes. These findings position DNA methylation as a critical epigenetic determinant of neural dysfunction, offering translational opportunities for targeted biomarker development and therapeutic intervention in alcohol‐related neurological disorders.

## Author Contributions

All authors contributed substantially to this work. **Fuyuan Deng:** conceptualization, data curation, formal analysis, investigation, methodology, software, validation, visualization, writing – original draft. **Junsheng Peng:** conceptualization, data curation, formal analysis. **Siran Lai:** conceptualization, data curation, formal analysis. **Guangpeng Zhang:** software, visualization. **Miaomiao Li:** software, validation. **Yuxin Huang:** investigation, methodology. **Mi Yuan:** conceptualization, methodology. **Gaolei Yao:** conceptualization, validation. **Peiming Zhang:** conceptualization, funding acquisition, methodology, project administration, resources, supervision, writing – review and editing. **Liming Lu:** conceptualization, funding acquisition, methodology, project administration, resources, supervision, writing – review and editing. All authors critically reviewed the manuscript drafts and approved the final version for publication.

## Funding

This research was supported by multiple funding projects, including the Peak‐Shaping Project under Guangzhou University of Chinese Medicine's Action Plan for Double First‐Class and High‐Level Disciplinary Development (GZY2025ZJ18), the Project of First‐Class Universities and High‐level Dual Discipline for Guangzhou University of Chinese Medicine (A1‐2601‐26‐415‐109Z67, A1‐2601‐24‐415‐109Z76, E2‐6399‐264‐109‐001, E4‐6401‐252‐109‐002), the Guangdong Provincial Higher Education Innovation Team Project (2025KCXTD011), the Shenzhen Medical Research Fund (C2501027), the National Natural Science Foundation of China (82405556), the Zhongshan TCM Heritage and Innovation Research Program (2024B3006) and the Postdoctoral Science Foundation General Project (2024M750464).

## Ethics Statement

As this Mendelian randomization analysis utilized publicly available summary‐level GWAS data, no additional ethical approval was required. The results will be disseminated through peer‐reviewed publication.

## Consent

The authors have nothing to report.

## Conflicts of Interest

The authors declare no conflicts of interest.

## Institutional Review Board Statement

Not applicable.

## Supporting information


**Table S1:** Details of the GWAS used in the study.
**Table S2:** The druggable gene.
**Table S3:** Instrumental variables for druggable genes.
**Table S4:** The result of MR analysis and sensitivity analysis for alcohol dependence.
**Table S5:** The results of colocalization analysis of alcohol dependence.
**Table S6:** The result of sensitivity analysis for alcohol dependence.
**Table S7:** The Steiger result of CDK5R1.
**Table S8:** The Steiger result of CAMKK2.
**Table S9:** The Steiger result of NRBP1.
**Table S10:** Replication analysis of CDK5R1 and alcohol dependence in GTEx (V10) database.
**Table S11:** Replication analysis of CAMKK2 and alcohol dependence in GTEx (V10) database.
**Table S12:** Replication analysis of NRBP1 and alcohol dependence in GTEx (V10) database.
**Table S13:** SNPS associated with the MR Effect of DNA methylation sites cg05102552 and alcohol dependence.
**Table S14:** The DNA methylation site cg05102552 and the MR results of alcohol dependence.
**Table S15:** The DNA methylation site cg05102552 and the MR results of NRBP1.
**TableS16:** SNPS associated with the MR Effect of DNA methylation sites cg07437263 and alcohol dependence
**TableS17:** The DNA methylation site cg07437263 and the MR results of alcohol dependence.
**TableS18:** The DNA methylation site cg07437263 and the MR results of CDK5R1.
**TableS19:** The enrichment results of SNP related to CAMKK2 in Finland.
**TableS20:** The enrichment results of SNP related to CAMKK2 in Finland.
**TableS21:** The enrichment results of NRBP1‐related SNPs in Finland.
**TableS22:** The enrichment results of SNPs related to the DNA methylation site cg07437263 in Finland.

## Data Availability

All data supporting the conclusions of this research are presented in the manuscript. The rest of the supporting information data are presented online (https://www.scidb.cn/s/eENFnu).
